# Current Insight of Printability Quality Improvement Strategies in Natural-Based Bioinks for Skin Regeneration and Wound Healing

**DOI:** 10.3390/polym13071011

**Published:** 2021-03-25

**Authors:** Syafira Masri, Mh Busra Fauzi

**Affiliations:** Centre for Tissue Engineering and Regenerative Medicine, Faculty of Medicine, Universiti Kebangsaan Malaysia, Kuala Lumpur 56000, Malaysia; eiramasri@gmail.com

**Keywords:** 3D-bioprinting, natural-based bioinks, wound healing, skin regeneration, 3D-printing quality

## Abstract

Skin tissue engineering aimed to replace chronic tissue injury commonly occurred due to severe burn and chronic wound in diabetic ulcer patients. The normal skin is unable to be regenerated until the seriously injured tissue is disrupted and losing its function. 3D-bioprinting has been one of the effective methods for scaffold fabrication and is proven to replace the conventional method, which reported several drawbacks. In light of this, researchers have developed a new fabrication approach via 3D-bioprinting by combining biomaterials (bioinks) with cells and biomolecules followed by a suitable crosslinking approach. This advanced technology has been subcategorised into three different printing techniques including inject-based, laser-based, and extrusion-based printing. However, the printable quality of the currently available bioinks demonstrated shortcomings in the physicochemical and mechanical properties. This review aims to identify the limitations raised by using natural-based bioinks and the optimum temperature for various applied printing techniques. It is essential to ensure maintaining the acceptable printed scaffold property such as the optimum pore sizes and porosity that allow cell migration activity. In addition, the properties required for an ideal bioinks design for better scaffold printability were also summarised.

## 1. Introduction

Skin injury has become a significant problem that can cause impairments to the patients’ quality of life [1]. A skin injury can be classified based on two different categories, which are acute and chronic wounds. An acute wound is usually able to recover within the wound healing time frame. There are several types of chronic wounds including wound infection, diabetic ulcer, and gangrene [2]. In 2018, Medicare beneficiaries identified 8.2 million patients with open wounds with or without infections in which this number is estimated to increase in the future [3]. In Malaysia, diabetic foot ulcers have become a significant concern among healthcare workers because of the prevalence of diabetes mellitus (DM) patients increases every year. These diabetic patients are prone to have chronic diabetic foot ulcers that are severe and involving a long-term impact on their lives [4].

Worldwide, diabetes has become a common disease with increasing cases daily. Based on the data reported by the National Diabetes Registry (NDR) by our Ministry of Health (MOH) Malaysia, the number of diabetic patients that have successfully registered by NDR was 1,614,363. This is targeted to increase in the future [5]. Furthermore, in the United States of America (USA), 6.5 million people are severely affected by chronic wound infections followed by an increasing number of diabetic patients with diabetic foot ulcers [6].

The National-Health Morbidity Survey (NHMS) reported that the prevalence of the diabetic burden in Malaysia increased from 15.2% in 2011 to 17.5% in 2015 [7]. The following statistics indicate that the prevalence of diabetes has increased approximately 14% within 5 years. An increasing number of diabetic patients reflects the increasing demand for wound-dressing supplies.

The Ministry of Health (MOH) Malaysia has a proper wound care guideline to handle wound injury. Wound care approaches are usually based on wound characteristics and assessments. Any wound exposed to infections will be prescribed antibiotics to stop the infection. Several types of wound dressing are available for wound treatment including hydrogel, hydrocolloid, alginates, foams, and films. The goals for each wound dressing are to maintain the wound’s environment, prevent infections, and minimise skin irritation [8]. Other than wound dressing, tissue engineering has been widely used and practised clinically to replace injured tissue due to chronic wound and promotes skin regeneration.

The application of tissue engineering has already been explored a long time ago using several conventional fabrication techniques. However, for chronic wounds, immediate treatment and tissue replacement are needed to avoid prolonged exposure to the environment. In skin tissue engineering, a 3D-shaped scaffold that has been seeded with cells is used to maintain the tissue homeostasis process [9].

A wound that is exposed to the environment is prone to get wound infections and complications. Therefore, 3D-bioprinting has been introduced to overcome the drawbacks of the conventional method especially related to production time. 3D-bioprinting has a high potential to deliver immediate treatment to the patient and plays a significant role in rapid treatment to promote skin regeneration and wound healing.

### 1.1. Cutaneous Micro Structure

Skin is the largest organ in the human body that can perform various functions including providing protection, controlling body temperature, and acting as a barrier towards physical, chemical, and biological hazards from the external environment [10,11]. Skin primarily contains a high protein known as collagen that functions primarily to maintain skin elasticity and promote the regeneration process [12]. Skin will undergo the hemostasis process immediately when there is any occurrence of a traumatic injury. Wounded skin is also known as a cutaneous wound. It needs to be treated immediately to avoid exposure to the environment that could lead to infections. Therefore, to achieve a complete skin regeneration, the cutaneous wound needs to undergo four stages of wound healing including hemostasis, inflammation, proliferation, and tissue remodelling [13].

Over several decades, skin substitution has been developed through skin tissue engineering technologies to construct and replace skin structures affected by chronic skin injury. The most common causes of skin injury include severe burns and chronic diabetic ulceration in DM patients. Chronic wounds that occurred due to severe burns and diabetic ulcers are mostly painful, hence they could impact the health conditions and may lead to disabilities [14]. Since the number of chronic wound patients increases, immediate treatment is necessary to avoid severe infections from the environment. Rapid wound healing is also crucial to maintain the normal homeostasis of the skin tissue [10].

The traditional wound dressing methods such as skin grafts that are considered as the standard gold treatment needed some improvements in technology advancement [13]. These technology advancements are being recognised through tissue engineering as an advanced initiative for wound healing and skin regeneration. In addition to wound healing, tissue engineering is also widely used in the pharmaceutical industry to replace drug delivery studies to observe dose-response and cancer analysis studies [15]. Skin tissue engineering involves the formation of bioscaffolds that mimic the native extracellular matrix (ECM) microstructure. Scaffolds can be developed using both conventional and advanced technology through the 3D-bioprinting approach. The conventional method of tissue engineering has several limitations in fabrication including insufficient pore sizes, closed pores, and slower fabrication processes [16].

3D-bioprinting is an advanced method in tissue engineering that can overcome the limitations of the conventional technique. It offers a faster fabrication process due to the 3D-printing technology [17]. The main component needed in the 3D-bioprinting is bioink, composed of a combination of biomaterials, crosslinkers, and cells. For skin tissue engineering, the types of cells needed are human dermal fibroblasts (HDFs), human epidermal keratinocytes (HEKs), and melanocytes.

**Figure 1:**
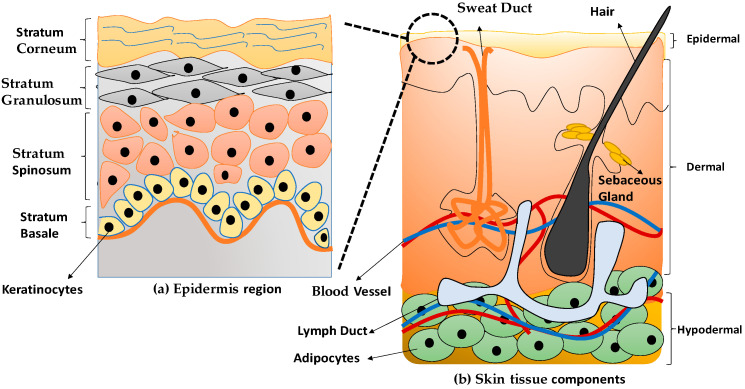
The anatomical structure of skin tissue (**a**) epidermis layer and (**b**) skin tissue components.

Keratinocytes are the main cell on the most superficial layer of skin known as the epidermal layer. The cells have a circular or spherical shape with approximately 80% of keratinocytes being deposited in the epidermis skin layer [18]. Figure 1a indicates the epidermal layer comprises four layers, namely stratum basal, stratum spinosum, stratum granulosum, and stratum corneum [19]. Keratinocytes tend to have a high differentiation and proliferation rate that mainly originated from the stratum basal layer. The cells can migrate towards the top surface of the epidermis layer to provide strength and protection to the skin. The dermis layer is considered the middle layer of the skin structure to support the epidermal layer. Moreover, the dermis layer also contains most blood vessels and nerves to distribute nutrition and gives sensation for the skin reflection [19]. Multi components of the skin’s layers explained in the Table 1.

Human dermal fibroblasts (HDFs) are differentiated from highly proliferative progenitor fibroblasts and responsible for maintaining skin tissues’ structural support [20]. During the formation of the dermal and wound repair process, HDFs are essential in response to stimuli and ECM proteins’ formation [21]. ECM also involves cells interactions, adhesion, proliferation, and differentiation to become a specific tissue [1]. Therefore, Figure 1 shows the anatomical structure of the human skin tissue.

### 1.2. Wound Healing Stages

Complete wound healing mechanisms usually involve four essential phases that are hemostasis, inflammation, proliferation, and tissue remodelling [24]. In wound healing progress, acute wound tends to settle in a shorter time with minimal complications compared to the chronic wound healing time [25]. In a chronic wound, the common problems encountered at the injury sites are insufficient blood supply at the lesion site, venous drainage, and infections [26].

**Figure 2:**
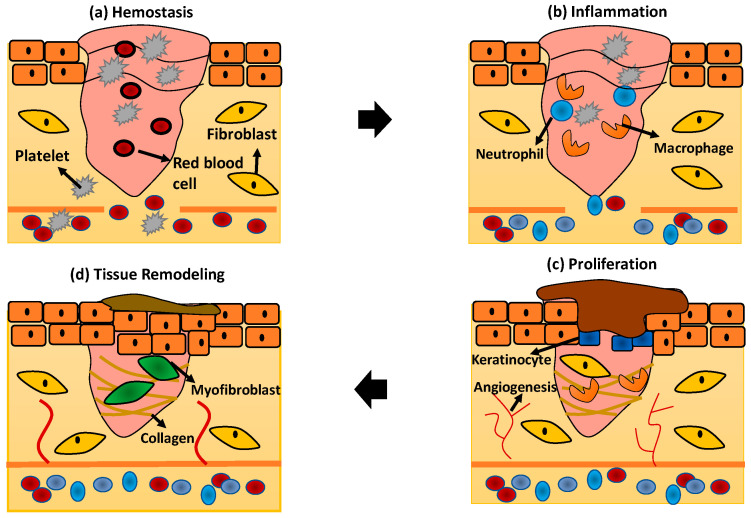
The wound healing phases (**a**) hemostasis; (**b**) inflammation; (**c**) proliferation; and (**d**) tissue remodeling.

Generally, every phase of wound healing will involve the activation of multiple cellular activities to promote skin regeneration. The schematic diagram in Figure 2 describes in detail the wound healing phases and skin regeneration mechanisms process:

#### 1.2.1. Hemostasis

The hemostasis process is the first response stimulated by the human body to stop the bleeding when the tissue and blood vessel become disrupted. In this phase, the blood vessel will immediately undergo vasoconstriction to prevent blood loss followed by primary and secondary hemostasis [25]. Primary hemostasis stimulates platelet aggregation to the injury site and interacts with the ECM protein components such as fibronectin, collagen, and factor VIII [27]. Secondary hemostasis will activate the coagulation cascade in which fibrinogen will be converted, forming a fibrin mesh at the injury site to trap red blood cells, and hence stopping the bleeding [25].

#### 1.2.2. Inflammation

The inflammation phase in wound healing is crucial referring to the response of the wound to the pathogen in the external environment. In response to infection at the injury site, the neutrophils, macrophages, and lymphocytes will be activated within several hours or days to perform phagocytosis [26]. The immune systems, especially neutrophils that play the main role in infiltrating the wound to remove pathogen by performing phagocytosis, will respond immediately [24]. After apoptosis by the neutrophils, the macrophages will replace the neutrophils to clear the debris and microorganisms. The macrophages will engulf all of the apoptotic neutrophils and release the inflammatory mediators such as TNF-α, interleukins (IL)-6, and IL-1β [27].

#### 1.2.3. Proliferation

The proliferation phase of wound healing focuses on the activation of skin cells including keratinocytes, fibroblasts, macrophages, and endothelial cells to proliferate and promote wound closure [27]. The formation of angiogenesis is incorporated in this phase to provide nutrients to the surrounding tissue. Macrophages are also responsible for activating the signal to enhance collagen production, smooth muscle cells, and other ECM components for re-epithelialisation [28]. The wound starts to contract for several days while actin and myosin start to pull the surrounded tissue to expedite the process of wound closure [24].

#### 1.2.4. Tissue Remodelling

After re-epithelialisation of the skin tissue occurred, the tissue starts to focus on the remodelling phase. The end product of tissue remodelling is usually a scab’s formation followed by the proliferation of the skin’s epidermal layer [14]. However, this phase usually lasted for 2 years for normal tissue recovery [24].

## 2. 3D-Bioprinting for Wound Healing and Skin Regeneration

Over the past two decades, tissue engineering technology has been widely used in medical applications to construct and replace the injured tissue. Tissue engineering technology offered therapies in skin regeneration and wound healing problems using 3D-bioprinting for skin tissue reconstruction that is previously associated with the conventional application method. An example of conventional methods is solvent casting and particle leaching, freeze-drying, gas foaming, and electrospinning that have demonstrated several drawbacks in fabrication techniques and time-consuming [29]. In addition, recent advancements in the current development of tissue engineering technology involves the use of 4-Dimensional (4D) bioprinting by transforming the shape properties of 3D-bioprinting such as folding and unfolding the printed scaffold [30]. The researchers are actively exploring the applications of 4D-bioprinting for in vitro and in vivo use in the near future.

The principle of replacing injured tissue with engineered-tissue has been developed by combining the provisional bioscaffold with cells and biomolecules including growth factors to the defect site followed by tissue maturation and remodelling [31]. Concurrently, with the development of the conventional method to produce scaffold, 3D-bioprinting becomes a new initiative with a combination of advanced technologies to promote better bioscaffold construction. The mechanism of action for 3D-bioprinting is to print the bioink layer-by-layer on the particular platform controlled by a computer-aided-design [16]. The imaging technologies via X-ray, computed tomography (CT) scan, and magnetic resonance imaging (MRI) could then be able to detect the anatomy and physiology of a defect tissue accurately [32]. It could be beneficial for the 3D-bioprinting approach by printing a specific dimension from the scanned images. The printed bioscaffold temporarily enhances cell regeneration and slowly degrades the time of tissue recovery.

In the last decade, 3D-bioprinting has become a practical approach in healthcare services to treat chronic diabetic ulcers and chronic wound repair. The 3D-bioprinting system successfully demonstrated that this technique enhances the healing process and promotes skin regeneration in the diabetic wound [33]. In addition, 3D-bioprinting also enables the use of technology with high advantages to fabricate the scaffold, mimicking the native tissue [17]. Hydrogel is the most favourable treatment in wound healing among researchers due to rapid wound healing progress. Therefore, the addition of human skin cells such as HDFs and HEKs in the hydrogels will further enhance the wound healing progress [34].

3D-bioprinting technology enhances the capabilities to produce a rapid fabrication process while controlling the scaffold porosity at an affordable cost with outstanding mechanical and structural properties of the bioscaffold [35]. The most prominent methods of 3D-bioprinting are magnetic bioprinting, stereolithography, photolithography, and extrusion-based bioprinting method [36]. The inject and extrusion-based bioprinting are the primary printing methods for fabrication technique in 3D printing due to their specific functions towards cells and bioinks used [37]. In the 3D-bioprinting field, two types of bioinks were used, namely natural-based and synthetic-based.

### 2.1. Current Limitations in 3D-Bioprinting Technology

The best criteria of physical and physiological characteristics of 3D-printed bioscaffold are to have a good shape fidelity, and this enhances cell viability after the post-printing process. However, natural-based bioinks have unique properties to develop scaffolds. The development of the bioinks needs improvement on the scaffold’s printability, physicochemical, and mechanical properties.

The selection of suitable bioinks for skin bioprinting is crucial in developing scaffolds that can support cells’ growth. The most common issues in 3D-bioprinting are selecting the suitable types of bioinks in skin bioprinting and optimisation of a suitable quantity of cells seeding for skin engineering [38]. Moreover, the main elements required in 3D-bioprinting technology include selecting polymer with low viscosity, stiffness, and cross-linking degree characteristics. It is essential to enable cellular activities including cell migration, nutrient transportation, and oxygen diffusion rate to promote the development of new tissue formation [39]. Therefore, the biomaterials used must establish scaffolds with good physicochemical and mechanical properties with better structural accuracy to support cells growth.

### 2.2. Bioinks for Skin 3D-Bioprinting

Bioinks are known as biological materials fluids loaded into the 3D-bioprinter system to construct scaffolds with a specific design using a layer-by-layer printing technique [20]. The main components involved to create the bioinks are biomaterials, cells, and suitable crosslinkers. Biomaterials have been invented with or without any extra modification to boost their functionality with cells in the human body [40].

Generally, bioinks must pose great functionality for a stable production of the biomimetic scaffold during printing [35] to avoid harming the printed human cells. In 3D-bioprinting, several factors need to be highlighted to achieve scaffold’s stability including physical, chemical, and mechanical properties. The physiological properties of the bioinks play an essential role in the development of printed tissue before reaching the maturation stages following time. Therefore, both the natural and synthetic bioinks are practically used in the tissue engineering field to construct particular scaffold designs to treat any defect tissues or organ.

#### 2.2.1. Natural-Based Bioinks

Natural-based bioinks are mostly non-toxic and have a favourable property. Most of the natural-based bioinks have good biocompatibility, faster biodegradation rate, non-toxic, optimum mechanical stability, maintain higher moisture content, and availability in wound management [38]. Additionally, the printable bioinks must support the printed cells to function normally and enhance ECM stimulation to resemble the native skin tissue microenvironment [14].

Several biomaterials derived from natural-based bioinks such as collagen, gelatin, alginate, fibrin, hyaluronic acid (HA), chitosan, and agarose have become the preference bioinks to fabricate the bioscaffold [41]. In the 3D-bioprinting field, gelatin and collagen are the most frequently used bioinks to fabricate the skin substitute due to their excellent physical, chemical, and mechanical properties [21]. Collagen is a triple helix structure protein that is present abundantly in the human body, especially collagen type I. Therefore, the use of collagen as bioinks are preferable and compatible with the printed cells. Furthermore, the biocompatibility of the natural-based bioinks compared to synthetic bioinks provides safer microenvironments to the cells. However, synthetic bioinks have advantages in constructing scaffolds with high printing fidelity and better mechanical strength [42].

In a previous study, several bioinks have been used by the researchers including collagen, gelatin, silk fibroin, and fibrin. These biomaterials derived from protein-based polymers are suitable for human use applications [43] since the essential elements resemble the native tissue matrices. However, the application of natural-based bioinks is limited due to low mechanical strength without combination with suitable crosslinkers. Crosslinkers are known as supportive elements that help to support the mechanical strength of the biomaterials. Besides, this review aims to focus on the printing limitation by using natural-based bioinks and possible strategies to overcome the weaknesses of the bioinks. Therefore, Table 2 shows the list of advantages and disadvantages of the natural-based bioinks towards skin tissue regeneration applications.

#### 2.2.2. Synthetic Bioinks

Synthetic biomaterials have been developed and explicitly invented with advance functionality in which a polymer is chosen to support several limitations of the natural-based bioinks, especially in enhancing the printed scaffold’s mechanical strength properties. Among the polymeric components of the bioinks, the common synthetic bioinks include polycaprolactone (PCL), polylactic acid (PLA), polyglycolic acid (PGA), polylactic-co-glycolic acids (PLGA), and polyvinyl alcohol (PVA) [43]. There are other types of synthetic polymers used as bioinks such as amphiphilic block copolymers, PEG, poly (PNIPAAM), and polyphosphazene [50].

Furthermore, several studies used a combination of natural and synthetic-based bioinks on selecting suitable bioinks for 3D-bioprinting. A study on the combination of PVA with gelatin showed improved water absorption ability and better mechanical strength with optimum biodegradation rate [51]. The combination of PLA as hydrophobic polymers with gelatin leads to better wettability properties while providing mechanical strength towards the soft gelatin hydrogel [52]. In 3D bioprinting, although the synthetic bioinks can produce scaffolds with high mechanical strength, most of the printed synthetic hydrogels lack cells’ active binding sites, thus creating an inappropriate microenvironment towards the cells resulting in low cell viability activity [42]. Table 3 listed the advantages and disadvantages of synthetic bioinks.

#### 2.2.3. Commercial Bioinks

Several commercial biomaterials have been introduced as bioinks for 3D-bioprinting such as ready to use bioinks of Dermamatrix, NovoGel, and CELLINK that have become the most popular commercial bioinks [53]. The production company will provide an application manual to the user and this increased the demand of commercial bioinks due to their simple application.

### 2.3. Type of 3D-Bioprinting Technique

Different types of printing methods have been introduced to enhance printability for different type of bioinks. The most common printability methods are the extrusion-based method, inject bioprinting, and laser printing. In extrusion-based printing, the diameter and morphology of the scaffold to produce well-defined 3D structures can be controlled using this technique [54]. This printing technique will apply mechanical pressure to push the bioinks through a nozzle. However, since 1990, the most widely used 3D printing method through extrusion-based method has been the fused deposition technique. The 3D printed products can be extruded under a high temperature of formulated inks to become solidified after printing [55].

In addition, inject bioprinting technique involves cost-effective technology and can print bioinks efficiently with higher printing rates compared to other printing techniques. Besides that, inject bioprinting can print a better structural composite bioscaffold with better cell viability than the extrusion-based bioprinting technique. However, inject bioprinting has a major limitation, which is printing high viscosity bioinks; this is due to their functionality [56].

Among all bioprinting techniques, extrusion-based bioprinting becomes the most appropriate technique to obtain a printed scaffold with higher mechanical strengths. Table 4 tabulated the limitations of different printing techniques with strategies to overcome printing quality.

### 2.4. 3D-Bioprinting Technique Phases

**Figure 3:**
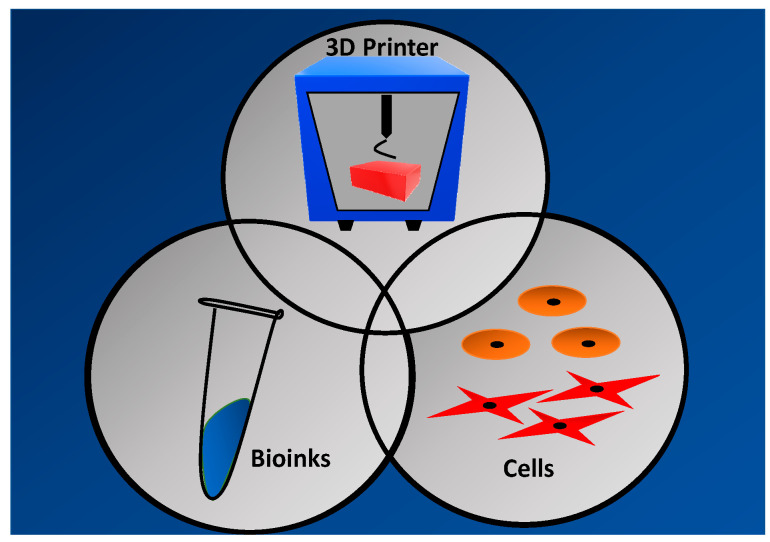
The elements needed for 3D bioprinting process.

A typical bioprinting technique involves three different phases namely pre-processing, processing, and post-processing [17]. A specific wound’s details will be obtained via wound scanner and transferred into the computer-aided-design (CAD) for further fabrication mainly in the pre-processing phase. The printing action of 3D-bioprinter includes selecting suitable biomaterials, type of cells, and ideal 3D-bioprinting technique need to be highlighted as a crucial part of the processing phase before developing a bioscaffold with good physicochemical and mechanical properties. Besides that, the maturation of the printed cells has been scrutinised before to in vivo implantation. Therefore, Figure 3 indicates the elements needed for 3D bioprinting process.

Furthermore, an ideal bioscaffold should provide an optimum tissue microenvironment mimicking the natural ECM to enhance tissue regeneration activity. The 3D-bioprinting technique comprises several options for the composition of cells and biomaterials (bioinks), printer properties, and conditions, including the type of printer used, optimum temperature, and oxygen level rate for the printing process [63]. An extremely high printer temperature may impede the growth of the cells. The usage of living cells for bioinks composition must include a sufficient growth factor to maintain the cell’s availability and promotes cells proliferation rate.

Despite the wide benefits of 3D-bioprinting, this technology faced some limitations including a lack of printed features and fabrication process [64]. Besides that, skin bioprinting is a challenge in designing suitable bioinks to fabricate 3D cellular bioscaffold with match skin geometries, magnificent shape fidelity, and high resolution of cells replacement activity [14]. Therefore, bioinks significantly need advance improvement to develop scaffold for skin tissue.

### 2.5. Limitations, Advantages and Prospects of Current Natural-Based Bioinks

3D-bioprinting can be described as using biomaterials to print various 3D printing modalities such as human organs or scaffolds [59]. Several factors can be highlighted to improve the printability of the bioinks including the gelation time for the hydrogel to polymerise after printing and the selection of suitable printing technique that can support the printability of certain bioinks. The printability quality of bioinks usually depends on the hydrogel’s optimum concentration, wettability properties, surface tension, ability to interact with crosslinkers, and the printer nozzle for the printing process [42]. All of these factors play a role to achieve a high-quality printed scaffold.

#### 2.5.1. Gelation Time for Natural-Based Bioinks

After printing, the first parameters that need to be observed are the gelation time for the printed hydrogel. The gelation time is incorporated with the flow of the bioinks throughout the printing nozzle. Therefore, it is essential to be monitored to prevent sedimentation of cells that can clog the nozzle of the syringe [65]. The formation of a clog in the printing nozzle will prevent the hydrogel from coming out from the syringe’s nozzle. In addition, the gelation time is related to the shape fidelity of the scaffold. Slower gelation time indicates low shape fidelity of the printed bioscaffold.

The gelation time varies based on the different types of bioinks. Natural-based bioinks such as collagen have a longer gelation time due to its low viscosity properties [66]. Increasing the viscosity of the hydrogel will increase the gelation time [49]. An increase in the gelation time can promote the structural fidelity of the hydrogel.

#### 2.5.2. Selection of Printing Technique

**Figure 4:**
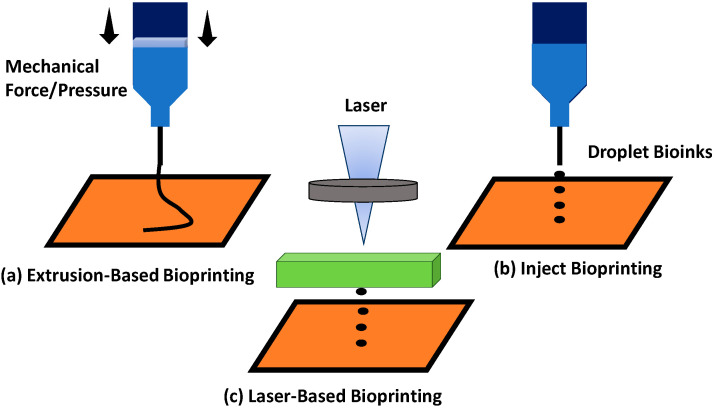
Different types of printing techniques: (**a**) extrusion-based bioprinting; (**b**) inject bioprinting; and (**c**) laser-based bioprinting.

There are three advance methods for 3D bioprinting including inkjet bioprinting, laser-based bioprinting, and extrusion-based bioprinting. All of these techniques have different advantages and also limitations towards the printability quality of the hydrogel. The principle of extrusion-based bioprinting is dispensing the bioinks into a syringe that can load the bioinks, and needles are attached to the syringe in which the hydrogel will come out through the nozzle via mechanical forces [37]. The syringe’s piston will be pressed to create pressure, hence releasing bioinks from the syringe needle, as seen in Figure 4a.

Besides that, the application technique for inkjet bioprinting is to load the bioinks into the syringe by applying a thermal or piezoelectric actuator [67]. The hydrogel will come out from the needle in the form of controllable droplet size to form a scaffold, as seen in Figure 4b. The laser-based bioprinting technique was also practically used for the fabrication of scaffold. This printing technique involves laser application deposit bioinks to produce the designed bioscaffold Figure 4c. However, this technique has disadvantages involving the high cost and the exposure of the laser, which might be too sensitive for the cells.

#### 2.5.3. Ideal Characteristics for 3D-Printed Bioscaffolds

The ideal characteristics of hydrogel have become the primary concern in 3D bioprinting because it needs to possess hydrated network properties that are important for gas exchange, nutrient transportation, and metabolite wastes removal for healthy cells and to promote cell viability [65]. The biomaterials must pose a characteristic that can be incorporated with living cells and to maintain the normal pH of the bioinks to ensure suitability for the cells. In addition, all biomaterials must have shear thinning properties because it is incorporated with printing difficulties. The shear-thinning properties of the hydrogels depend on the viscosity of the bioinks. The hydrogel must have the ability to heal after facing shear stress during printing.

## 3. Factors That Affect Low Printability Quality in 3D-Bioprinting

The 3D-bioprinting technique is very challenging due to its printing issues that affect the scaffold’s printability quality. The printability can affect the gross appearance, morphology, and mechanical properties of the scaffold [68]. Several factors can influence the printability quality of 3D-bioprinting including the type of printing method, type of bioinks, the viscosity of the hydrogel, shear-thinning property, scaffold porosity, and structural fidelity. All of these printability factors are summarised in Table 5.

## 4. Strategies to Achieve Optimal Printability Quality for 3D-Bioprinting

Physicochemical properties of the printed scaffold are incorporated with the interactions of biomaterials with the living cells. The physicochemical properties that were most commonly highlighted to obtain a suitable bioscaffold includes the viscosity of the bioinks, shear-thinning property, scaffold porosity, and structural fidelity.

### 4.1. Shear-Thinning Properties

Shear-thinning properties have become important factors that need to be considered to achieve a good printability goal. The shear-thinning of bioinks is divided into three stages. Firstly, the bioinks should be able to flow through the printing nozzle, indicating the shear-thinning behaviour of the bioinks [70]. The yield stress shows the amount of force needed to initiate the flow of the bioinks.

Generally, the shear-thinning behaviour is closely related to the extrusion-based bioprinting technique. It helps in obtaining printing fidelity with a stable mechanical strength for the hydrogels to support the cells’ growth and to perform normal functionality [41]. The gel state bioinks will face low shear stress during the printing process. The shear force starts to develop when the bioinks start to polymerise during printing [47]. The ability of the bioinks to flow through the nozzle depends on their viscosity. However, the rheological test for bioinks still lack standardisation in the parameters to determine the shear thinning properties.

**Figure 5:**
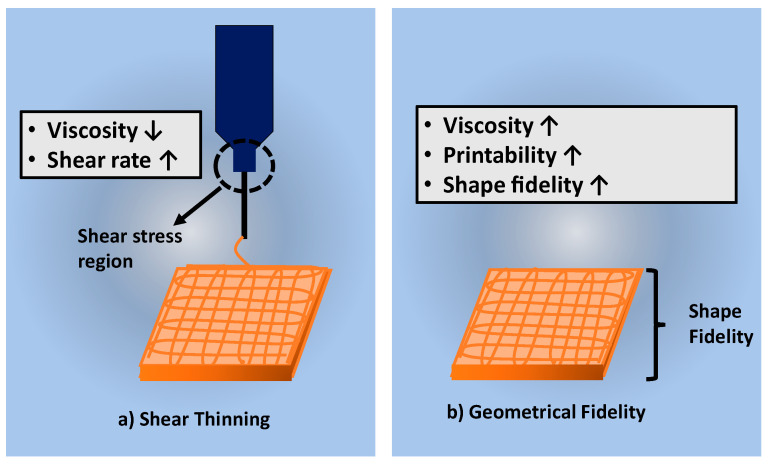
Shows the factors affected the printing process (**a**) shear thinning and (**b**) geometrical fidelity of bioscaffold.

Table 5 discussed the shear-thinning properties of the hydrogel. The cells suspension in the bioinks may be affected by the developing shear stress due to higher hydrogel’s viscosity [60]. High mechanical force or pressure needs to be applied to push the high viscosity hydrogel out from the nozzle. A previous study on the shear-thinning properties of gelatin-elastin bioinks by using extrusion-based bioprinting indicated that the level of shear stress increases when the pressure in the extrusion piston increases [72]. Figure 5a shows the shear stress region in the syringe during the printing procedure. The shear stress will usually clog the cells to sediment at the bottom of the nozzle causing difficulty in printing out the hydrogel through the nozzle. Therefore, bioinks need to be designed to have shear thinning property to overcome the shear stress and the surface tension that occurred during printing [64].

In order to allow the hydrogels to come out from the printer’s nozzle, the bioinks must possess shear-thinning properties as a push factor to the printing technique. However, a study on alginate bioinks reported to have high viscosity, and thus will induce shear-thinning properties during printability by using extrusion-based bioprinting [73]. Therefore, the printability quality of alginate bioinks can be achieved using a suitable nozzle size and adjust the viscosity based on a suitable concentration for the printing process [44].

Another study on the furfuryl-gelatin bioinks developed low shear stress while using extrusion-based bioprinting due to the low viscosity of the gelatin. This indicates that the potential of cells viability using gelatin bioinks also increases. Based on the discussion above, we can conclude that the shear-thinning properties of the bioinks depend on the viscosity of the bioinks that may vary according to the different concentration of bioinks. It is a challenge to achieve the accuracy of bioinks. Many studies reported that the nozzle diameter and extruder rate of the bioinks can help in determining the shear stress [74]. Therefore, to improve the shear-thinning behaviour, several factors need to be adjusted including the bioink’s concentration, temperature, and total cells density for the printing process [70].

### 4.2. Structural Fidelity of Bioscaffold

In 3D-bioprinting, shear-thinning properties and geometrical fidelity are incorporated between each other to produce hydrogels with excellent mechanical strength. The structural fidelity of a bioscaffold is vital to maintain the shape of the bioscaffold after printing technique. The high-fidelity structure of hydrogels is influenced by the shear viscosity of the bioinks [54]. In the extrusion-based bioprinting technique, the high viscosity of the bioinks resulted in a high shape fidelity [54]. However, Figure 5a shows contradicting shear-thinning properties. The high viscosity of bioinks will result in a low shear-thinning rate.

Recent strategies for maintaining the shape fidelity of the printed scaffold are through the combination of biomaterials with the crosslinkers. The crosslinking technique can be broadly divided into physical and chemical crosslinking methods. Physical crosslinking involved interaction between the polymers by forming ionic bonds while chemical crosslinking involved permanent and irreversible covalent bonds [74]. Therefore, hydrogels need to be combined with other materials to support their shape fidelity.

Hydrogels contain high water content compared to other scaffolds. Therefore, the printing technique becomes challenging because it will disrupt the shape of the composite scaffold, low printing accuracy, and difficulty in obtaining a highly porous bioscaffold structure [60]. For example, alginate bioinks tend to have low structural fidelity due to its low viscosity. To overcome this limitation, the addition of gelatin and honey into alginate bioinks strengthened the printed hydrogel structure with adjustable viscosity [69,73]. Alginate bioinks are also suitable to be crosslinked with the physical crosslinking method to enhance structural fidelity [74]. Besides that, a study on collagen bioinks indicates that collagen has low viscosity properties [61,81,82]. Therefore, the collagen needs to be incorporated with agarose to enhance the mechanical structure stability, improve the viscosity, and help to increase the gelation time [83].

However, the shape fidelity of the hydrogel can be improved by printing a complex structure of the hydrogels [72]. The main concept of 3D-bioprinting is to print the hydrogel layer-by-layer until it forms a composite scaffold. The shape of the hydrogel needs to be adequately designed before printing and being concerned about the shape fidelity factors. The combination of bioinks can support the structural fidelity of the hydrogels. A study on the combination of silk fibroin with other polymers has reported having a better shape fidelity after printing [65].

The low viscosity of bioinks gives low shape fidelity of the hydrogels. A study on the combination of alginate bioinks with honey as a natural remedy enhances the shape fidelity since honey has a high viscosity level [84]. Alginate tends to have a low viscosity level compared to honey. The suitable range for honey concentration to be used with alginate has been highlighted and tabulated in Table 5. A structural fidelity study on gelatin bioinks also has been conducted by the researchers. Gelatin bioinks were not able to support the structural fidelity of the hydrogels due to high water content and soft structure. Therefore, gelatin has been used with nanocellulose to keep the printed hydrogel’s shape fidelity [75]. Therefore, the limitations of the printed hydrogel’s shape fidelity can be overcome by adjusting the viscosity of the bioinks, printing shapes, and the use of crosslinkers or other polymers.

### 4.3. Optimum Viscosity of Bioinks

Viscosity affects the flow of the bioinks through the printing nozzle. The viscosity of the bioinks is related to the shear stress action that occurred in the nozzle. For extrusion-based bioprinting, the high viscosity of hydrogels will face difficulty to flow out from the printing nozzle. The high viscosity of bioinks will cause the cells to start to sediment at the bottom in the bioinks, clogging the nozzle or needle of the syringe. Therefore, a study on the optimum bioinks viscosity suggested that ideal bioinks must possess low viscosity to prevent clogging in the nozzle [85]. Besides that, low hydrogel viscosity also will protect the cells from damage that may be caused by fluid shear stress.

**Figure 6:**
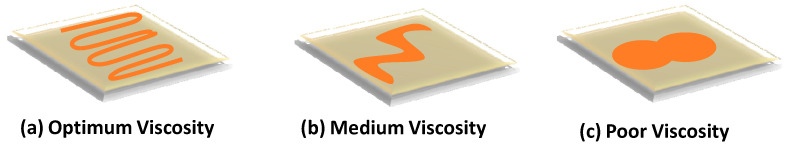
The level of printing viscosity for bioinks: (**a**) optimum viscosity; (**b**) medium viscosity; (**c**) poor viscosity.

Figure 6a indicates the optimum viscosity of the bioinks in which the shape of the printing can be designed and appropriately achieved during the printing procedure. In contrast, Figure 6b indicates that the medium viscosity of the bioinks started to lose printing shape compared to Figure 6a. Figure 6c shows poor printing shape caused by too low hydrogel viscosity.

Many natural-based bioinks have been widely used for bioprinting due to their suitable viscosity properties. Alginate is known as one of the natural bioinks that is most commonly used due to its rapid gelation time and adjustable viscosity according to different concentrations [21]. However, the low viscosity of alginate bioinks will interrupt cell viability [74]. In addition, alginate bioinks have been successfully proved to be safe for in vitro and in vivo study application, although the high viscosity of the hydrogel may alter the pore size of the hydrogels.

A study on the collagen bioinks tends to have low viscosity properties that will affect the hydrogel’s printing quality. The fabrication process using collagen bioinks is more complicated than other bioinks because of its low viscosity and rigid control during printing. Collagen becomes part of our ECM that functions to support the structure of the cells and is, therefore, safe to be used for skin tissue engineering and wound regeneration applications [50]. The gelation time for collagen bioinks is much slower than other bioinks due to their low viscosity characteristics [82,83]. Therefore, to overcome the limitation of the low viscosity of the collagen bioinks, the collagen needs to be used together with agarose to obtain the optimal viscosity of bioink [83].

Bioinks must possess high viscosity properties to support the printed hydrogel’s essential characterisation, which maintain the shape fidelity. Gelatin is known as a natural-based bioink that has a reverse effect on gelation or polymerisation properties. The gelatin’s viscosity is usually incorporated with temperature during printing [72]. Therefore, to overcome the limitation of the gelatin’s low viscosity, the concentration of the gelatin needs to be increased and in use in combination with other natural bioinks such as alginate or with an additional crosslinker to allow high printing fidelity [73,74,75]. The discussion on the strategies to achieve optimum bioinks viscosity is based on suitable concentrations, temperature, and depending on the type of crosslinkers used to overcome the limitations of bioinks with low viscosity properties.

### 4.4. Highly Porous Scaffold

Living cells need a porous structure scaffold to promote cell spreading through the scaffold and allow cell migration activity through the scaffold’s interconnected pores [54]. Besides that, the optimum pore size of the bioscaffold also enables drug delivery actions. Previous studies reported that the use of natural-based bioinks gives an excellent scaffold porosity product. A study of alginate scaffold porosity revealed that the alginate scaffold’s pore sizes are in various sizes ranging from 5–200 nm according to different concentrations [44].

Although alginate has become the most widely used bioink in 3D-bioprinting, the fabricated scaffold tends to have a lower porosity structure. Therefore, alginate was combined with chitosan to form a highly porous scaffold with high cell viability [83]. The printability of the collagen bioinks is more stringent than other bioinks due to its limitation in gelation time. Besides that, collagen bioinks are not able to produce a highly porous structure due to a low level of viscosity and mechanical properties. The low viscosity resulted in a complex crosslinking procedure.

Overall, the high porous structure of the scaffold is very crucial to allow cell migration towards the pores. The highly porous structure will enable ample nutrients and minerals absorption for the cells.

### 4.5. Biodegradation

In tissue engineering, biodegradation can be defined as the ability of the scaffold to degrade or break-down after being implanted in the human body. Scaffolds need to degrade to ensure that the surrounding cells are safe and non-toxic. In 3D bioprinting, researchers usually use hydrogel to fabricate a composite scaffold prior to application in humans, since it is degradable. The biodegradation test needs to be performed to identify the effectiveness of the scaffold after implantation into the real human tissue. Therefore, bioink must possess an optimum rate of biodegradation to ensure the normal function of the cells.

Bioinks derived from natural-based polymers usually possess good biodegradability properties. Generally, collagen provides mechanical strength, eligible pore size structure, and a high biodegradation rate [83]. However, the biodegradability study of collagen bioinks towards in vivo applications is under evaluation for future use. Hence, in some cases, collagen bioinks need incorporation with synthetic polymers to support the biodegradability rate [50]. This is because synthetic polymers have a low biodegradation rate. However, in skin tissue engineering, the combination of collagen with gelatin bioinks provides a better printability quality for skin regeneration activity, since this combination produces a better quality of bioscaffold for patient’s skin [1].

In addition, certain natural-based bioinks degrade at a slow pace, hence it depends on an enzyme for a faster degradation rate. For example, alginate lyase, known as an enzyme, was added into the mixture of alginate and gelatin to enhance the degradation rate and promote the cells’ cellular activity [86]. The enzyme will boost the degradation rate when in contact with human tissue since the human body lacks an alginate lyase enzyme, thus this factor is very crucial for alginate biodegradation. Besides that, in 3D bioprinting, alginate has been extensively used as a bioink because it can create cell-friendly environments, although it undergoes the gelation process [87].

The biodegradability rate of the 3D printed scaffold is also related to the percentage of the oxidation received and has been classified into three different levels including poor degradability (0% oxygen level), moderate degradability (5% oxygen level), and high degradability (10% until 15% oxygen level) [28]. Biodegradation properties are essential for the scaffold to be applied to the human skin tissue.

## 5. Conclusions and Future Perspectives

In summary, the 3D-bioprinting technique has become an advanced method for treating wound healing and skin regeneration. There are two types of bioinks that are available to be used for 3D-bioprinting, namely natural-based and synthetic-based bioinks. Natural-based bioinks have been widely used in the 3D-bioprinting field because it is non-toxic towards human tissue; having an optimum biodegradation rate; and having a tendency to construct a bioscaffold with excellent physicochemical and mechanical properties. However, several limitations affected the printability quality of the natural-based bioinks such as different printing techniques, shear-thinning properties, the viscosity of the selected bioinks, scaffold porosity structure, and structural fidelity of the bioscaffold. Each bioink has different limitations and a unique application technique that needs to be applied to enhance the scaffold’s physical, chemical, and mechanical properties. Therefore, this study has successfully revealed the limitations of the printability in 3D-bioprinting with strategies to overcome printing limitations. This review discussed the designation of the natural-based bioinks to print a better hydrogel to focus on human skin regeneration and wound healing. In the future, we recommended the use of natural-based bioinks with suitable printing techniques in in vitro and in vivo studies, with a variety of printing temperatures to observe the effect of cellular activity of the cells.

## Figures and Tables

**Table 1:** The layers of the skin with multi components and cell types.

Layer of Skin	Components of Skin Layers	Cell Types
(a) Epidermal	Composed of stratum corneum, stratum granulosum, stratum spinosum, and stratum basal [18,22]. Made up of stratified squamous epithelial tissue [22].	Keratinocytes, melanocytes, Langerhans cells, and merkels cells [17,22,23].
(b) Dermal	Sebacous gland, sweat gland, collagen/elastic fiber, nerve, hair follicle [18,22]. Made up of two regions; papillary dermis and reticular dermis [22].	Fibroblasts, smooth muscle cells, endothelial cells, Langerhans cells, fibrocytes, lymph vessel [18,22,23].
(c) Hypodermal	Blood vessel (artery and vein)	Adipocytes or fat cells [22]

**Table 2:** The list of advantages and disadvantages in skin regeneration applications for each of the different natural-based bioinks.

Natural-Based Bioinks	Advantages	Disadvantages
Alginate [1,44]	Alginate has hydrophilic properties with high viscosity and provide suitable environment for living cells to grow	Alginate hydrogel is too watery. Thus affecting the mechanical stability of the printed hydrogels. The viscosity features of alginate can heal skin tissue damage and promote cells proliferation rates.
Collagen [1,45,46,47]	Collagen can easily found in human and animal with fibrous like structures. Known as biocompatible biomaterial and suitable for supporting tissue adhesion and proliferation. Collagen type I mostly abundant the in human body and support the growth of the skin dermis layers.	Collagen type I will stimulate cytokines reaction such as inflammation and sometimes can cause damage to the skin tissue.
Hyaluronic Acid (HA) [1,48]	HA has hydrophilic properties. It can combine with water due to the speciality of the biochemical structure.	HA has pro-inflammatory and pro-angiogenetic properties.
Chitosan [1,49]	Composed of d-glucosamine and *N*-acetyl-d-glucosamine. Chitosan has an excellent combination with other natural bioinks.	Less stable if use alone as bioinks. Less solubility in an aqueous media due to its semi-crystalline polymer characteristics.

**Table 3:** The list of the advantages and disadvantages of skin regeneration applications for synthetic bioinks.

Synthetic Bioinks	Advantages	Disadvantages
Polyvinyl Alcohol (PVA) [1,21,47]	PVA show a great combination with other crosslinkers. Have excellent physicochemical properties, including biocompatibility, non-toxic polymers, resistant towards chemical, hydrophilic polymers, and optimum biodegradation rate. Have excellent mechanical strength.	PVA needs higher temperature to be dissolved, thus will affect the growth of cells. Take a longer time to be dissolved in distilled water.
Polyethylene glycol (PEG) [1,46].	PEG have excellent application in skin tissue regeneration because it is not involved in the skin vascularization process. Improve features of bioscaffold.	Have low cell affinity.
Polylactic acid (PLA) and poly-L-lactic acid (PLLA) [1,46,47]	Have excellent biodegradability and biocompatible properties for skin regeneration.	Not suitable for high temperature application.

**Table 4:** The summary of limitations, advantages, and strategies to overcome printing quality of different types of bioprinting methods.

Type of Printing Method	Description of Printing Technique	Limitations	Advantages	Strategies to Overcome Printing Quality
Inkjet Bioprinting [47,52,57]	Involve printing of bioinks in the form of droplets: piezoelectric and hot-bubble type.	Cause cell death due to thermal damage.	Low cost of printing technique. Simple technique applied and provided faster printing speed with good cell viability.	The ink viscosity needs to be adjusted within a suitable concentration to avoid the nozzle from clogging due to its smaller diameter size. Use piezo inkjet heads. It does not involve thermal and can control the liquid droplet formation with a wide range of nozzle sizes.
Laser-Based Bioprinting [47,56,58]	Involve absorption of laser and the heat will be transferred to become a gas with high pressure form.	It also involves a high-cost technology with a time- consuming procedure. UV light can cause cell damage.	Cells can maintain the normal function and perform the cellular activity because they are not exposed to any mechanical stress and can print out the high viscosity of bioinks.	Use visible light to enhance polymerization rate. The visible light will not cause harm to the viability of the cells.
Extrusion-Based Bioprinting [37,47,56,58,59,60]	Involve gas compression, piston mechanical forces action, and screw type technique for delivering the bioinks through the pump.	Pressure involved during printing can cause cell damage.	Suitable to print out various type of biomaterials and involve the low cost of printing technology.	Use high viscosity of bioinks. Optimize the bioinks ratio and viscosity before printing to achieve high mechanical strength of the printed hydrogel.
Stereolithography Bioprinting [47,56,61]	Use ultraviolet light.	Ultraviolet light cause cell damage.	Efficient and easy to control printing technique.	More research on how to overcome the limitations of stereolithography bioprinting is still actively explored by the researchers. For instance, the photocrosslinking process and resolution need to be controlled during the printing method.
Microfluidic Bioprinting [47,56,62]	Micro-on-a chip	Not able to entirely print human skin structures. It is difficult to maintain the precision of the hydrogel during printing.	High efficiency and low cost. Low shear stress applied during the printing process.	Use a single step fabrication process to improve the printing quality and workflow to print the tissue/organ.

**Table 5:** The factors that were affected by low printability quality in 3D-bioprinting technique.

Bioinks	Printing Method	Factors that Affected by Low Printability Quality				Strategies to Improve Printability	References
		Viscosity of Hydrogel	Shear-Thinning Property	Scaffold Porosity	Structural Fidelity		
Hydrogels	Extrusion-based bioprinting Lithography-based bioprinting	Higher viscosity of the hydrogel will result in high printing fidelity.	Shear stress increases due to high viscosity of hydrogels.	The thickness of the hydrogel layers may influence the size of the pores.	Cross-linker efficiency and structural stability for postprinting.	The optimal temperature of each hydrogel must be identified because it has influenced viscosity of the hydrogels. Increase printing resolution for shape fidelity. Hydrogels must be physically or chemically crosslinked to facilitate the shape of the 3D-structure. Several printing patterns were suggested to enhance pore structures, including zigzag and honeycomb patterns.	[60,69]
Alginate-Gelatin	Extrusion based bioprinting	High viscosity of alginate-gelatin bioinks promotes unstable and irregular forms of hydrogels during printing. The viscosity of the alginate-gelatin bioinks is influenced by the temperature of the gelatin to become gel and solid. The higher viscosity of gelatin will result in higher modulus storage. Besides, the higher viscosity of alginate will increase in loss modulus.	Not-Reported	Not-Reported	Alginate and gelatin have low structural fidelity. Loss modulus of the alginate will negatively affect the shape fidelity of the printed hydrogel.	The concentration of gelatin must be higher than alginate to ensure right viscosity and storage modulus. The optimum printing temperature for alginate-gelatin is between 20–25 °C. Alginate known as low bioadhesivity bioinks. Therefore, alginate need to be used with gelatin to provide the ligands for cell attachments and mimics the native ECM. The covalent crosslinking technique should be used to enhance the mechanical properties of alginate. The printability quality of alginate-gelatin bioinks can also be supported by the addition of an extruder heating system.	[69,70,71,72,73,74]
Agarose-Collagen	Extrusion-based bioprinting	Collagen has low viscosity and slow gelation time. Agarose has rapid gelation time and its viscosity influenced by the temperature.	Not Reported	Not Reported	Agarose supports the mechanical strength of the collagen bioinks.	Collagen type I needs to be used with agarose to enhance the viscosity, gelation time, and support the mechanical strength. The strategies to improve shear thinning and porosity structure for agarose-collagen bioinks are not reported.	[69,75]
Chitosan-Gelatin	Extrusion-based bioprinting	The viscosity increased as the concentration increases.	Flow rate increased according to the diameter of the nozzle	Chitosans have shear thinning behavior.	Chitosan-gelatin hydrogel has excellent mechanical strength.	Appropriate concentrations of the chitosan-gelatin bioinks should be used since they have influenced the viscosity of the hydrogels. The optimum size of the nozzle is necessary to monitor the printing of the hydrogel. Chitosan must be combined with other natural biomaterials for better mechanical stability.	[69,76,77]
Cellulose-Alginate	Extrusion-based bioprinting	A lower viscosity of alginate will disrupt cell viability.	Not Reported	Not Reported	Not Reported	The combination of alginate with nanofibrilated cellulose (NFC) resulting an excellent 3D printing.	[69]
Silk fibroin-Gelatin	Extrusion-based bioprinting	The viscosity of silk fibroin influenced by the temperature.	Exposure of shear force >100 s^−1^ towards silk fibroin bioinks during printing results in nozzle clogging.	Have interconnected pore structures that enable cellular migration activity.	Printed hydrogels that are made up of silk have high compatibility with high structural fidelity.	Mix homogeneous living cells before printing process to allow easy mixing and achieve optimal viscosity without affecting cell viability. Apply low shear force (<100 s^−1^) during printing to reduce shear rate. The printed hydrogel can be deposited in 80–90% of alcohol to permit a faster solidification. However, this is not suitable with cells. Silk fibroin need to combine with gelatin bioinks to produce putative cell attachments motifs.	[49,65,69,78,79]
Gelatin-Elastin	Extrusion-based printing	The viscosity of the gelatin-elastin bioinks depending on the adjusted temperature.	Shear stress increased from 0.79 to 1.17 kPa when the extrusion pressure increased from 5 kPa to 25 kPa	Not-Reported	Construct with a complex architecture shape of the scaffold will improve the printing fidelity.	Handle with a temperature of 8 °C for optimum viscosity. The final printing condition was selected as 15 kPa pressure and 30 mm s 1 at 8–10 °C, resulting in 1.08 kPa shear stress. Used cold water fish gelatin to enhance the printability of bioinks. Crosslinking with visible light is required to enhance the mechanical strength of the hydrogel. Strategies to enhance porosity structure for gelatin-elastin hydrogels are not reported.	[71,72]
Alginate-Honey	Extrusion-based bioprinting	The use of alginate alone tends to be high in viscosity and therefore difficult to print.	High viscosity of alginate induces shear thinning during the printing process.	Alginate hydrogel has low porosity structure.	Low shape fidelity.	Use honey as natural materials/remedies to reduce the viscosity of alginate, improve the structural fidelity of the printed hydrogel, and increase the gelation time. Use up to 5% concentration of honey to retain the porous structure of the printed hydrogel. Strategies to improve shear thinning for alginate-honey bioinks are not reported.	[73]
Alginate	Extrusion-based bioprinting	The viscosity of alginate bioinks influenced by the amount of alginate powder and suitable temperature use.	Not Reported	High porosity of hydrogel structure.	Not Reported	Choose the right size of nozzle/valve for printing because it affects cell viability and shear thinning rate. Alginate bioinks suitable to perform physical crosslinking to enhance shape fidelity.	[44,74]
Gelatin Methacrylate (GelMA)	Extrusion-based bioprinting	The adsoption of GelMA towards nanocellulose has impacts on the viscoelasticity of the hydrogel and it becomes easier for the hydrogel to move out from the nozzle.	Nanocellulose shows shear-thinning behavior.	Not Reported	The incorporation of GelMA with nanocellulose increased the solid content of the bioinks. Therefore, it will increase the shape fidelity of the hydrogels.	Adjusted the printing parameters based on viscoelasticity of bioinks. Used 2000 mm/min of printing speeds. Combine GelMA bioinks with nanocellulose to enhance mechanical strength of the hydrogel.	[80]
Furfuryl-Gelatin	Extrusion-based bioprinting	Insufficient viscosity for printing.	Insufficient shear thinning.	Have adequate porosity structure for cellular activity.	Low structural fidelity.	Addition of a small quantity of hyaluronic acid (HA) to enhance the viscosity of the hydrogel. Strategies for managing shear thinning are not reported. Requires crosslinking with visible light to achieve good structural fidelity.	[81]
Collagen	Extrusion-based bioprinting	Low viscosity	Increase in shear rate	The usage of collagen bioinks without a crosslinker does not produce a porous structure of hydrogel.	Weak mechanical strength.	Use of low pH, mild collagen composition showed dense collagen fibers with a large pore size. Print collagen bioinks below gelation time (35 °C) to prevent shear stress. 5% collagen is the optimum concentration to reduce shear stress and for high cell viability. Crosslink the collagen bioinks with a crosslinker (physical or chemical), or can use with other biomaterials including natural and synthetic polymers to enhance mechanical strength of the hydrogels.	[82,83]

## Data Availability

The data presented in this study are available on request from the corresponding author.
